# The Etiology of Southern Cattle Plague—Texas Fever

**Published:** 1892-11

**Authors:** Frank S. Billings

**Affiliations:** Director of the Patho-Biological Laboratory of the State University of Nebraska


### THE ETIOLOGY OF SOUTHERN CATTLE PLAGUE—TEXAS FEVER.

(*Concluded*).

By Frank S. Billings,

*Director of the Patho-Biological Laboratory of the State University of Nebraska.*

### THE MANURE OF SOUTHERN CATTLE THE CHIEF MEDIUM BY WHICH THEY INFEST OUR NORTHERN PASTURES AND THROUGH WHICH OUR NORTHERN CATTLE BECOME INFECTED.

In the preceding pages the fact that the Southern cattle plague is caused by a bacillus has been completely demonstrated. It has also been shown that that same bacillus is the fœcal discharges of ticks taken directly from Southern cattle, as well as in the internal fluids of the ticks. The same bacillus was also found, not only by plate-cultures, but by the inoculation of a guinea-pig in the manure of one of the calves, into the jugular vein of which a pure culture of the bacillus from the tick had been injected. These unquestionably demonstrated results should indicate the chief manner, not only by which Southern cattle infest our Northern pastures, but also, if the ticks do play any part in the drama of this American cattle plague, that it must also be through sucking the infested blood from Southern cattle and by their own (the ticks') discharges, also infest our Northern pastures. Though there are some mysteries in the reported investigations of the Agricultural Department, especially in the case where the young ticks which were artificially hatched from ova and placed on Northern cattle, said to be so placed that there was no possibility of their deriving the disease in any other way than through these young ticks, still we must ever bear in mind two facts :

1st. The long-continued and well-proven unreliability of all such statements emanating from that source.

2d. The absolute improbability, practically an impossibility, of the tested cattle ever having derived Texas fever in the manner claimed, especially when we also take into consideration the admission of the Agricultural Department that while "these brief notes demonstrate that Texas fever can be produced by placing young ticks on cattle, and that the disease cannot be due to any abstraction of blood, for the ticks are quite small and have scarcely begun to draw blood on a large scale." (Report of Bureau, 1889–90, p. 98, l. c.).

I say, with that admission and the other improbabilities of this etiological fairy tale, there must have been some other factor at work of which the investigators of the Government make no mention.

That that factor could have been the fœcal discharges of Southern ticks goes without question. That it could have been the same of Southern cattle is also equally plain. That it could have been anything else is not probable and scarcely possible.

It will be remembered also that we have no mention of a single attempt having been made to test either the blood or organs of any of the cattle mentioned, or the ticks, for any micro-organisms whatever, which shows most blamable neglect and a totally unscientific method in conducting these investigations. It will not do to "come at me" after these words are published with the

*a priori* assertion that such investigations were made. I know those persons better than that. They may have been, it is true, but had they fallen out negative as regards the presence of micro-organisms, the circumstantial evidence of their malignant desire to injure the work of this laboratory is of so positive a nature that no unbiassed observer will believe for a moment that they did result negatively, and no mention made of the details of proof and how it was obtained.

Quite the contrary is warranted by all the facts connected with the department investigations.

Those people in Washington are "very clever," as our English cousins say; "very sharp," as we Americans express ourselves. This had been exemplified in many ways, which, while looked over in some circles of society, certainly are unpardonable in a department of Government created by the people for their own benefit. The accidental presence of a micro-organism of the same species of the German swill-milk disease in hogs, mistakenly called "Schweine-seuche," was a most fortunate "find" for the Government investigators, and the most "sharp" practices resorted to force a second widespread "swine plague" on this country, as an offset to the growing appreciation for the Nebraska investigations, especially in the endeavor to show the futility of inoculating against the true swine plague alone. This "trick" having failed to win, the next thing we experience is the wolf-cry of the terrible dangers of inoculation, which will eventually have the effect that the "wolf" did on the bad boy. Now we have the tick protozoon hypothesis, if we dare to honor so absurd a statement even with that word, as the cause of the Southern cattle plague, hoping thereby to again nullify the value of our work in the minds of the stock-raisers of this country.

It is singular how fortunately events sometimes turn out for people of that character. The Loeffler-Schutz work gave them the foundation for a second swine plague, and the accidental discovery of a similar germ which could only be surely discovered by rabbit inoculation gave them the necessary powder. Lauerans and others' work supplied the protozoon; Koch supported it, and either the *a priori* assumption of its presence in the red blood cell of Southern cattle, or some process of necrosis of the plasma of that cell (it is a well-known fact that the cells disintegrate in this disease), gave color to the assertion.

Another most accommodating circumstance was the real correspondence in the cycle of development from old ticks, through ova, to young ticks, the time required corresponding very closely with the period averagingly elapsing between the arrival of Southern cattle on our Northern pastures and the eruption of the disease in native cattle.

They tell us that "it requires from forty to sixty days for the matured ticks to drop from Southern cattle and the eggs laid by them to develop into young ticks" (l. c. p. 97), a most fortuitous circumstance, which fitted very nicely into the results of my previously published investigations.

OUTBBEAKS OF TEXAS FEVER IN THE NORTH DUE TO SOUTHERN CATTLE.

| | Place and date of arrival of Texas cattle. | Date of outbreak. | No. of days after arrival of Southern cattle North. |
|---|---|---|---|
| 1. | Warren Co., Ind., June 12, 1868 | August 4, 1868 | 52 |
| 2. | Warren Co., Ind., June 12, 1868 | July 29, 1868 | 46 |
| 3. | Odell, Ill., June 25, 1868 | August 10, 1868 | 47 |
| 4. | Northfield, Ohio, July 4, 1868 | Sept. 1, 1868 | 57 |
| 5. | Farina, Ill., May 10, 1868 | July 15, 1868 | 65 |
| 6. | Tolona, Ill., June 25, 1868 | July 28, 1868 | 33 |
| 7. | Sodoro, Ill., June 1, 1868 | July 28, 1868 | 56 |
| 8. | Champaign, Ill., June 15, 1868 | August 3, 1868 | 49 |
| 9. | Tekomah, Neb., April 1, 1887 | July 1, 1887 | 90 |
| 10. | Roca, Neb., June 29, 1887 | Sept. 1, 1887 | 63 |
| | | | 558 |

Average period between date of arrival and first notice of disease in Northern stock, $55\frac{80}{100}$ days.

It is at once to be seen how closely this average corresponds to the average time for the cycle of development in ticks to occur as stated above. On the other hand, while I positively know nothing about it, it does not seem exactly reasonable to suppose that in the cycle of tick development we should have the extreme variations shown in the outbreaks of the disease following the arrival of Southern cattle on our Northern pastures. It does seem to me altogether impossible for the tick-ovulation protozoön theory to conform to the actual facts occuring in this disease, while the fæces of both cattle and the ticks on them, lodging the germ and protecting it so far as cattle manure is concerned, are conformable to the entire phenomena of the development of this disease in Northern stock. While I cheerfully admit that the Southern ticks direct from the cattle can possibly supply fæces enough charged with the bacilli to play some part in causing this disease in Northern stock, that they do not play any essential role in any way seems supported by the one peculiar clinical phenomenon in this disease, which is, that it has never been known to extend across, under or over a wire fence to cattle on the other side, though killing those in the infested pasture with wondrous rapidity. This fact requires some exact observation with reference to the ticks. I have seen such an outbreak when the apparently unaffected cattle in an infected pasture were actually touching hair and licking cattle in an adjoining field; I have myself made an autopsy on a steer which in dying fell against the wires and was half through such a fence, yet the disease never went to the other cattle. I know the diseased cattle had some ticks on them. The trouble is I did then think to study the tick question on the other side of the fence, not being much inclined to "straddle a fence" on any question. Still it does seem to me as if under such circumstances ticks should pass from the cattle in one field to those in the other, and that did the ticks play any essential part in the etiology of this disease, that sometimes it would have passed the boundaries of a wire fence. Had it ever done so we should certainly know it in this country.

THE SALIVA–URINE THEORY IN THE ETIOLOGY OF THIS DISEASE.

The hypothesis that the bacilli of the Southern cattle plague were chiefly dispersed on our Northern grazing grounds by means of the salivory drewlings and urine of Southern cattle has been most strongly set forth by Dr. H. J. Detmers in the report of the Department of Agriculture for 1884. These media of infesting our pastures need no serious consideration, as neither of them have consistency enough to protect the bacilli against the sun's rays and other unfavorable influences. Were they the acting media of transplantation we should see infection following almost immediately on the advent of Southern cattle in the North. That this has never occurred in less than thirty-three days, so far as we know, and generally requires over fifty, has been conclusively demonstrated. With the mature-tick-ovulation artificial hatching protozoön invasion process we have nothing to do, having completely exploded it. We have then to consider in more detail.

THE MANURE OF SOUTHERN CATTLE AS THE VEHICLE OF INFECTION.

As already shown, the bacillus of this disease was in the fæcal discharges of the ticks and the inoculated calf. Were the germs not in the manure of the Southern cattle when brought to our Northern pastures the danger of Southern cattle plague in our native stock would be comparatively nothing, for even though we may admit that the fæces of the ticks do contain the germs, they do not offer the necessary protection to them which the vicissitudes of the weather make necessary to preserve their votality. That it must be through their manure that the Southern cattle chiefly infest our Northern pastures is most strikingly exemplified in the Tekomah outbreaks. Up to the time of this outbreak and another at the same time due to Southern cattle shipped at the same time and from the same place into Illinois which also caused an outbreak in that State, the period of safety in which Southern cattle were permitted to be shipped North for grazing purposes was fixed at March 31st, it being supposed that up to that time the weather was cold enough to prevent any danger to Northern stock. The general idea has always been that "the first frost put an end to any danger of infection to Northern stock. The experiences at this time completely exploded that idea and the time at which importations must cease was put back to either March 1st or February 1st, I do not know which at this moment, and it matters not in the case, as I only desire to show that we had most severe freezing weather after April 1st, 1887, and yet it had no effect on the future outbreaks of the disease, and to show also that it must have been the manure which protected the germs, and hence the outbreaks. On the shipment of cattle into Illinois at the time mentioned, the following letter is very instructive :

"*Dear Sir*—Yours to our Mr. McChesney is at hand, also a copy of the "*Nebraska Farmer*" with your very interesting article on " Texas Fever" which we carefully read. We at once tried to learn the facts about the shipments of Texans, as mentioned by you, into this State. We found that a large lot were received from the region of country named, but could not learn if they came from the same parties. These cattle were a mixed lot, Texans and Shorthorn grades. A part of them were sold and taken from the yards March 30th to a farm twelve miles away. During this time the weather was cold, ice being formed at night. They were driven to Kankaku county. On the first farm they stopped over night Texas fever appeared July 16. The native cattle were turned into the pasture used by the Texans May 10th. Several died and post-mortems made by our veterinarians established the fact of Texas fever. All along the track to point of destination the same disease appeared. The owner states that none of the Texas lot have been sick.

"Very respectfully yours,

"Jno. M. Pierson,

"Chairman Live Stock Com'n."

The exact correspondence in every detail with the results at Tekomah, Neb., the same year, must be apparent to every one who has read these pages carefully. The 1,100 Southern cattle arrived at Tekomah, Neb., March 30 and 31st, and every one remarked that it froze hard every night for two weeks subsequently, which fact was frequently quoted to me, and has since been repeatedly talked over as having given the people a feeling of perfect safety as to any dangers to their native stock from the Texas cattle. As said before, every cattleman in the North believes that that "the first frost would kill Texas Fever," but no one had thought that "first frost" which had always been noticed in this connection came in the Fall of the year to be followed by constantly increasing cold weather, and not, as in this case, in the Spring with warm weather rapidly approaching, and the sun undoing the work of the frost every day.

Had the germs been bound on the saliva or urine of the Texas cattle and not destroyed by the frosts, the disease must have broken in some of the native cattle much earlier, as it only takes about twenty days to cause it after a replete development of the germs in the pasture has taken place.

Something must have protected the germs from the outward action of the frost during the cold nights of those two first weeks of April, 1887. What was it? It could have been nothing else than that they locked up in the manure of the Texas cattle dropped in pads on the pasture. So long as the pad of manure dropped by infected cattle—Texans or natives—remains moist, soft and cohesive, there is no danger to native cattle placed on pastures where such infected cattle have been. The results at Tekomah demonstrate that fact with positive certainty. The second outbreak emphasizes it much more sharply than the first; for the climatic conditions were favorable all the time between the first and second outbreaks —from July to October—yet there were only twenty days' actual variation in the time in which the eruption took place if we carry the first outbreak to a time when climatic conditions made infection possible in native cattle.

The merely freezing of the surface of the manure pads in the early days of April helped put them in a condition favorable to the preservation of the germs within them. It made a hard, close crust on the outside of the pad of manure, which was also increased by the evaporation caused by the ever increasing heat of the sun in the daytime. These two factors together also materially aided in increasing and supporting the temperature of the inner portions of the pad, thus forming the life and increase of the germs. A careful comparison on plates made between the number of specific bacilli in the old pads of manure and those in the fresh manure taken directly from the large intestine of a killed animal demonstrates a far greater number of the Southern cattle plague germs in the old pads in comparison to the intestinal manure. That these germs will keep their vitality for a long time in such manure when protected from evaporation and extreme temperature variations is demonstrated by the fact that in the summer of 1887 I took some of the moist contents of the inside of one of these pads dropped by the Texas cattle and tested it then, as above, with fresh manure, and again in March, 1888, and March, 1889, both by plates and tests on ground squirrels, and even to the last time the germ did not seem to have lost in virulence in the small animals. Thus it will be seen that as the time elapsed and these pads of manure became older, they must have become an almost dense culture of the specific germ, and when in May, by means of the elements, they had become broken up and distributed in the grasses of the fields, a most general infection must take place as soon as the warmth was sufficient, the growing grass protecting them from the direct action of the sun. Only after the pasture had been thus prepared were the natives thereon—the twenty-one cows—really exposed to infection, and not before, no matter how long they may have been there previously. This accounts for the long time, ninety days, elapsing between the arrival of the Texas cattle at Tekomah—April 1—and the first outbreak in natives—July 1, 1887, and explains why the frost did not kill the germs.

In the second outbreak, where the twenty-four native steers infected their own pasture, leading to the infection of their companions in September, the same cycle of events had to be passed through. The twenty-three infected cattle were in and of themselves not the least dangerous to their 114 companions, which had remained in the pasture while the others were wandering around and confined in the pasture infected by the Texans, though for one night only. They, as cattle, were not dangerous. This fact is recommended to those investigators who insist that "contagious," in the hygienic sense, is the same thing as transmission by inoculation. The Southern cattle plague is transmissible by inoculation. They were vehicles for the conveyance of a vehicle, their manure, in which was the specific cause, the bacillus of the diseases from the pasture infected by the Texas cattle, to their own, in the same manner as the Southern cattle were also vehicles of the same vehicle, and the same germs from the grazing grounds of Texas to those at Tekomah. The manure of those twenty-three infected cattle was the shell of the bacillary-dynamite which was later on to explode in their 114 companions But when this manure was first dropped by twenty-three sick natives it was not dangerous to the 114 others, or we should have had infection in twenty days, as in the twenty-three which were confined in the thoroughly prepared (to infection) pasture, where the Texans had been from April 1st to 15th. It was not dangerous so long as the germs were confined in the solid and broken pad of manure as dropped by the infected cattle. As before, the germs were constantly developing in the pad, and directly from the time it was dropped, in a most rapid manner, because the climatic conditions were all the time favorable. There were no cold, frosty nights and cool weather to check the operation. The drying up of the pads also took place much more rapidly, and there were 114 cattle in the pasture walking through them and distributing the manure in small fragments, aided by the elements, in the roots of the grasses, thus innocently preparing the way for their own funeral. Not until a general dissemination of the germs had thus taken place was there any danger to the 114 other natives. They grazed on these death-secreting grasses in blissful ignorance of the wrath to come, which occurred in about fifty-three days from the time their twenty-four companions were returned among them from the Texas-infected pasture.

This time appears unnaturally long when we consider that the climatic conditions were far more favorable to the rapid development and dispersion of the germs in the grasses than in the first instance, but my observations teach me that the germ loses some in virulence in passing through cattle, and that it gains it again gradually when developing in earthy material. The Southern cattle came North with their intestines freshly loaded with the germs from their native soil ; they remain in these cattle some two weeks before their intestines become thoroughly cleared out of their Southern food, as shown conclusively by the experience at Tekomah, where the Texans arrived March 30th and 31st, 1887, and every place they went previous to April 15th became afterward pestiferous to natives, while the places where they were subsequent to April 15th were perfectly harmless to native cattle. The tests made with the germ from my inoculated steer taken from the Roca cattle, both in the first generation, showed a loss in virulence in the inoculated steer in comparison with the first culture from the Roca animals directly infected from the pasture infected by the Texans. With these remarks I think that the bacillus of the Southern cattle plague has been most exactly demonstrated, as well as that the manure of Southern and infected native cattle is the chief medium for the transference and protection of the germs by which our pastures are infected, and cattle infected with this, the most virulent and malignant of American cattle diseases. It has also been shown as conclusively, I think, that if ticks do play any essential part in the etiology of this disease, that it must be the mature tick coming North on the Southern cattle which suck the germ-infected blood from the Southern cattle and pass the bacilli off in their fæces to the ground, and not the young ticks hatched from ova after the arrival of the Southern cattle in the North.

### NATURE OF THE SOUTHERN CATTLE PLAGUE.

In considering this question it will be impossible to avoid the repetition of some things which have previously been said in the consideration of the etiology of the disease, but there seems to be such a vast amount of ignorance on this subject, such a complete neglect of sound reflection and every logical sequence in the minds of many investigators and mere practitioners, that such repetition is not only pardonable, but demanded. To decide correctly as to the primary nature of a given disease seems to be a most complicated question to many, whereas with competent education, acute powers of observation, and logical, conclusive abilities it should be a very simple matter.

The pathology of infectious diseases naturally differentiates itself into two distinct yet directly related fields of observation.

1st. The primary origin of its cause, the causa sufficiens, the inficiens proper.

2d. The direct and indirect action of that inficiens in the infected organism; the pathological anatomy, the primary and secondary lesions.

With the second, the pathological anatomy, we shall have little to do at this time, as it has been treated in the necroscopical notes detailed in the etiology. Again, the Southern cattle plague is so acute an infection that the pathological anatomy presented is generally a very clear picture, unclouded by secondary complications, which renders that stock much easier than in the study of diseases of a slower type and hence much more liable to secondary complications, such as the swine plague and corn-fodder disease.

Regarding the nature of the Southern cattle plague in relation to its primary origin and differentiation from other infectious diseases in that respect, we shall also deal quite briefly, on account of the fact that various causes necessitate a most detailed consideration of that subject in connection with actinomycosis, or "Lump Jaw," in cattle, which will be the subject of the next bulletin on cattle diseases published from this laboratory.

Every well-read and reflecting student of medicine, particularly such as have studied works of hygiene, published at any time previous to the past decenium, well knows that the generally accepted expression of the writers was and still is, though we use other terms to express our conclusions:

"That while all contagious diseases are infectious, that all infectious diseases were not contagious."

Thus was and is a known and universally admitted difference indicated.

Many modern writers, but especially a small array of unintelligent imitators, have endeavored to deny this absolutely practical differentiation, by asserting that the words contagious and infectious were identical in meaning. I do not care an iota as to the sanctity which antiquity is often assumed to give to a statement or conclusion, any more than I do to the weight a great reputation is often assumed to give to a so-called authority. Positively and without reserve, I am my own authority, and so is every other man who has any opinion worthy of a moment's consideration. Infallibility is by no means claimed in that statement. We are all liable to error. Nevertheless, after weighing all the evidence, our own conclusions must be our infallible guide in all matters. The great trouble is that the majority of the human race have no evidence of their own by which to weigh that of others. They blindly accept the "has been" because of the supposed sanctity of age and its authors. Those who can observe acutely the phenomena of nature and draw conclusions that will stand the test of time have been and still are rare productions in the march of human progress. Whether right or wrong in my conclusions time will alone tell. One thing is sure, that is, that as every other man, I have no other light than that of my own intelligence to guide me. The work of others can be but a reflected light by which each of us may strengthen himself in the pursuit of knowledge. Unfortunately, only too many find the greater portion of their intellectual light in the transmitted reflections of the past. As the heat of the sun of yesterday aids that of to-day in developing our growing harvest, so the reflected intellectual lights of the past add warmth and strength to our own endeavors; but as yesterday's sun also adds strength and vigor to the weeds which we cut down and they dry and wither in to-day's sun, so the light of our ever increasing intelligence must cut down the weeds in the transmissions of past authorities and cause them to wither on the wayside of human progress. He who does this lives to-day, though he dies to-morrow. He is no disrespector of the great authorities of the past. Their reflected light is the oil which makes the lamp of his own intellect burn all the brighter. The wick of past authority constantly needs trimming. Only the lamp of universal truth, filled droplet by droplet with the oil of knowledge, drawn from nature by the laws of investigation, burns constantly on the altars of human advancement. The flame has never yet gone out, though at times the wick has become thickly crusted with accumulated ignorance, or bitter errors, due to the sanctity which unobserving man has had for dead authority. But still the flame flickered and gave some warmth, and anon comes an Aristotle, a Hippocrates, a Galileo, a Luther, a Boerhaave, a Hunter, a Bichat, a Newton, a Darwin, a Virchow, a Koch, a Pasteur, a Franklin or an Edison, and with one fell stroke the crust of superstitious reverence for authority is knocked heedlessly to one side, and again the lamp of truth burns and its warmth radiates through the nations, once more replenished with fresh droplets of the oil of knowledge wrung from nature's fountains by the intellectual giants of a living present.

Such has been, is, and will continue to be, the history of the medical as well as all other branches of human knowledge.

Beware! Let us not bow down in holy reverence before any "strange god" erected by our own ignorance and assert he and he only is an authority. Every man must be his own authority unto himself. Otherwise the world would die of intellectual dry rot ere another ten years had passed over our heads.

It is this "intellectual dry rot," this respect for and leaning on some other man's light rather than our own, which is the cause of all this confusion as to the natural classification of infectious diseases. I use the word "natural" purposely. All artificial classifications are but "intellectual dry rot." Read the history of medicine and the great artificial "platforms" on which diseases have been piled up in classified order in the past, if any reader doubts Science is not art. Learn that lesson once for all and you will learn the first great principle necessary to become an investigator of natural phenomena. Science is method. Science is the methodical study of nature's methods, in order to apply them to human necessities. If the result be artificial it fails. If it be a simple, direct repetition of nature it succeeds and becomes a beacon light to further research in the same direction. The method of application is the art, but not the method itself. If the method is natural, then success follows. If artificial, it is unnatural, and failure, partial or total, results. That is a repetition, but not an unnecessary one. "Look more to nature than to the authorities" should be the rule of all true students. That is the inductive method which has led to all our modern acquisitions of knowledge, and the gradual adaptation of natural forces to our necessities. The deductive method may be said to be the "weeder" that subjects the remitted results of the past to the living sun of the present. Only the true grain stands the tests.

With a constant eye on the records of the past we as constantly press forward in the search of nature's truths.

The differentiation of the past that "while all contagious diseases are infectious, all infectious diseases are by no means contagious," indicating two great classes, had and has to do with the primary origin of all infectious diseases only, and not with their clinical course, or the relation existing between healthy and diseased individuals of a susceptible species.

The "contagious diseases" of earlier writers are now distinguished as "obligatory parasitic" or "endogenous" diseases—that is, diseases in which the specific cause, the inficiens proper, invariably finds its source, so far as we have any historic record of the fact, in some one species of animal life, the chemismus of which's tissues is such as to offer the essential elements necessary to the life of the germs of such diseases.

Examples: Syphilis, contagious pneumonia, rinderpest, the spotted typhus, and the specific eruptions of man, scarlet, measles and other diseases of like origin.

The diseases named above have also this peculiarity, that their possibilities of natural transmission are entirely limited to the species of life in which they primarily occur. There is another class of endogenous obligatory parasitic diseases which are not thus closely limited to one distinct species, but are naturally transmitted, generally by accident, to one or another species. Such are glanders, rabies, the foot and mouth disease of cattle, and variola. More of this class are found in the domestic animals than in man. More animal diseases are possible of transmission to man than diseases of the human species to animals by natural accidents.

The susceptibility of the individuals of any species of animal life to the action of any specific inficiens is determined in both fact and degree by the favorable or unfavorable qualities of the nutrition offered the specific germs by the chemismus of the exposed or infected organism. Where the chemismus is unfavorable, no infection results. The pathogenic quality of micro-organisms is, under natural conditions, far more decided by the chemismus of the exposed organism than by any quality of the germs themselves, though the latter cannot be entirely disregarded, for certain unknown, known, ascertainable or experimental influences can so alter the character of specific germs that for the time they may be unable to "manufacture" toxic or disease-producing principles from the food offered them by certain organisms, but frequent and repeated transmissions through such organisms, or experimentally in certain food, will result in the germ in question acquiring its pristine virulence, or in some cases incurring it, while in others it is decreased, and finally lost altogether. It is now an admitted fact that the specific germs of non-recurrent disease produce both a toxic, or disease-producing, and an immune-producing, or preventive, chemical principle. It is also becoming evident that the so-called "curative," or resistant principle, by which infected individuals survive, is the result of the action of some specific organ of the body, which on irritation produces a greater quantity, and that this chemical product of the infected organism unites with the toxic product of the germs and thus forms a new and non-irritating or non-disease-producing substance. When this product and combination takes place with sufficient rapidity, so-called recovery results. It is a sort of modern return to the dyscrasis and crasis theory of Hippocrates with an entirely different application. It is still an open question, to my mind, if all forms of germ, as every form of life, do not also produce another chemical product which in and of itself is eventually capable of being the cause of the death of its own producers, and which, when we can produce and isolate it in sufficiently concentrated quantities can be also of use as a therapeutic agent with much more certainty than the antagonistic product of any organ of the infected organism.

The protective-preventive vista of pathogenic investigation is only dawning on us at present, but the promised possibilities are most encouraging.

FACULTATIVE-PARASITIC OR EXOGENOUS INFECTIOUS DISEASES.

Directly opposed to the obligatory-parasitic or endogentic diseases are the facultative-parasitic or exogenous diseases in their primary origin. That is, diseases in which the causa sufficiens or inficiens proper as invariably finds its primary origin in conditions offered by the external surroundings of animal life as the endogenous causes do in those offered by the animal organism itself.

Here again the relation between diseased individuals and healthy ones of susceptible species the possibility of transmission has no bearing whatever on the differentiation. Here, again, nutrition decides the point of infection, but with this difference—the germs find their natural conditions favorable to their existence in external conditions, soils, etc., but where any species of animal life also offers a similar chemismus the germs have the faculty of living in their bodies, when introduced, and becoming specifically pathogenic ; that is, disease-producing.

We must distinguish most sharply between naturally pathogenic and experimentally pathogenic, as has been pointed out in another place. The investigators of the Agricultural Department have done their utmost to confuse the farmers of the country on the points here discussed, using the word contagious constantly in reference to swine plague, which is a plain facultative-parasitic or exogenous disease, and not a contagious one as asserted.

The mystifying language has been used in connection with the Southern cattle plague, where they say :

"There is no doubt that it is a difficult matter to understand how it is possible for the native cattle of a section permanently infected with a contagious plague to resist the influences of the contagion with which they are surrounded. It is equally difficult to understand how apparently healthy cattle can distribute contagion for so long a time after they leave the infected districts. It is equally difficult to understand why the cattle really sick (Northern) of this contagious disease do not convey the contagion to others." Report 1883, p. 20.

The careful reader of these pages should easily understand how all these things occur. In the first place, a more striking example of a facultative-parasitic or exogenous disease, hence not contagious—does not exist. The transmission is through and by the manure of the infected animals and in no other way. It is easy enough for an educated and competent intelligence to understand why the Southern cattle do not themselves show manifest signs of disease, though it is now admitted that many of them do die of it in their native country. John Gamgee said:

"In the South splenic fever is distinctively indigenous, and so far as Texas is concerned, I have satisfied myself that the disease is universally prevalent in the State." Report on cattle diseases, 1871.

Again: "What are the active causes in operation which tend to influence the stamina of Southern herds? Traveling over the prairies no one can fail to be struck with the large number of dead animals to be met with. The dissection of these animals, or the slaughter of the first animal met with, reveals their distinct and unfavorable manifestations." (Ibid, p. 107.)

Gamgee conclusively shows that Texas cattle do have the Southern plague; though the majority do not die of it, and it is a part of that majority which comes north to us. That they do not die is due to the fact that they take in the germs in small doses from the time they begin to graze while sucking calves, a period when all herbivora are less susceptible to diseases of this character than at any other time in their lives, and before they are harmed they have acquired immunity against further disease-producing powers in the germs, with which still their bowels are completely filled and which they bring north with them when imported, and infect our pastures as already fully explained.

There is nothing difficult to understand in the whole matter to any person having a healthy and active understanding.

The influence of Laverans observations in fever and ague, and Koch's conclusions as to malarial diseases, necessitates that we again briefly define

WHAT A MALARIAL DISEASE IS.

It is an exogenous facultative parasitic disease of a most local character, being neither naturally transmissible with any species of animal life, nor transportable from its place of primary origin by a diseased person, or any secretion or excretion from such a diseased individual.

It is self-evident that the Southern cattle plague is not a disease of such a character. Its local origin in the soil of certain sections of the South is as marked as is the transportability of its inficiens by cattle from such localities to our Northern pastures, and the follow-infection of our native cattle.

How native cattle can be the means of extending the disease further to a second lot of natives, has been fully explained. All that is necessary is that the original lot of Southern cattle come North early enough to guarantee a prolonged enough existence of suitable temperature for the full order of events to occur in the manner described.

As a pathological entity, a study of the necroscopical notes accompanying this paper, and the clinical symptoms of this disease are sufficient to demonstrate to any competent person that the Southern cattle plague is a specific septicæmia; a blood-poison due to the development of a specific irritans—the bacilli—into the blood from the intestines, and the nutrition of, by the blood of the bovine organism, being favorable, the germs thereby secrete, reproduce the toxic or disease-producing principle which gives to the disease its specific characteristics in its clinical course and pathological phenomena.

This is no new idea, as the same conclusion was arrived at by the investigators of the Metropolitan Board of Health of New York, and published in the transactions of the New York Agricultural Society in 1868, p. 1109.

